# Anatomically revealed morphological patterns of pyramidal neurons in layer 5 of the motor cortex

**DOI:** 10.1038/s41598-020-64665-2

**Published:** 2020-05-13

**Authors:** Siqi Jiang, Yue Guan, Shangbin Chen, Xueyan Jia, Hong Ni, Yalun Zhang, Yutong Han, Xue Peng, Can Zhou, Anan Li, Qingming Luo, Hui Gong

**Affiliations:** 1 Britton Chance Center for Biomedical Photonics, Key Laboratory for Biomedical Photonics of Ministry of Education, Wuhan National Laboratory for Optoelectronics-Huazhong University of Science and Technology, Wuhan, 430074 China; 2 HUST-Suzhou Institute for Brainsmatics, JITRI Institute for Brainsmatics, Suzhou, 215100 China

**Keywords:** Motor neuron, Neural circuits

## Abstract

Neuronal cell types are essential to the comprehensive understanding of the neuronal function and neuron can be categorized by their anatomical property. However, complete morphology data for neurons with a whole brain projection, for example the pyramidal neurons in the cortex, are sparse because it is difficult to trace the neuronal fibers across the whole brain and acquire the neuron morphology at the single axon resolution. Thus the cell types of pyramidal neurons have yet to be studied at the single axon resolution thoroughly. In this work, we acquire images for a Thy1 H-line mouse brain using a fluorescence micro-optical sectioning tomography system. Then we sample 42 pyramidal neurons whose somata are in the layer 5 of the motor cortex and reconstruct their morphology across the whole brain. Based on the reconstructed neuronal anatomy, we analyze the axonal and dendritic fibers of the neurons in addition to the soma spatial distributions, and identify two axonal projection pattern of pyramidal tract neurons and two dendritic spreading patterns of intratelencephalic neurons. The raw image data are available upon request as an additional asset to the community. The morphological patterns identified in this work can be a typical representation of neuron subtypes and reveal the possible input-output function of a single pyramidal neuron.

## Introduction

Neural cell types are one of the fundamental information for understanding the neural system from a structure perspective. Neuron types can be defined anatomically, molecularly, or physiologically^1–3^. However, neuron types categorized based on molecular or physiological data may be a biased representation of neuron types. Because the molecular and physiological techniques have their own limitation that the system usually cannot acquire data across the whole mouse brain and the data are usually available only for a local area^4,5^. Meanwhile, the anatomy research can provide complete long-range information of an individual neuron. Thus, neuron anatomy can be used for cell typing given its unbiased view of neuron morphology across the whole brain. While it is possible, it is still labor intensive to acquire complete neuron morphology at the single axon resolution and trace fibers across the whole mouse brain. Previous researchers have mainly studied pyramidal neurons in the local area at higher resolution^4^ or across the whole brain at a lower resolution^6^. For example, pyramidal neurons in layer 5 of the mouse motor cortex, whose functions are often studied in brain circuit analysis, spread their axons across the whole brain^7^. Thus, it is critical to reconstruct and analyze the anatomy at the single axon resolution across the whole brain to define the cell types for this case.

Before the whole brain neuron fiber scale image acquisition system is available, researchers cannot acquire sufficient neuron samples for anatomy study at the single axon resolution in a comprehensive whole brain image dataset. Brain slices have been the commonly used method to identify an axon projection pattern. Through this technique, researchers^6,8–10^ focus on the axons originating from the mouse motor cortex and projecting to particular brain nuclei without quantification data of an individual neuron. According to the previous research^1,11^, pyramidal neurons in the mouse brain can be divided into five types based on their projection pattern: local projecting layer 4 neurons, intratelencephalic (IT) neurons, pyramidal tract (PT) neurons, cortico-thalamic projection neurons, and layer 6b subplate neurons. It is now possible to acquire a whole brain single axon resolution imaging dataset for neuron morphologies^12–17^ with the development of optical whole brain imaging system such as fMOST or STPT. Using the neuron reconstruction technique of whole brain at the single axon resolution, Economo *et al*.^7^ classify pyramidal tract (PT) neurons in the mouse motor cortex into two subtypes anatomically for the first time. Lin *et al*. have reconstructed several intratelencephalic (IT) neurons in the secondary motor cortex of a mouse^18^, whose projection patterns vary both in morphology and projection pattern. Thus it is important to reconstruct more neurons of single neuronal fiber resolution with the cytoarchitecture information in order to systematically quantify and analyze the fiber projection patterns of the pyramidal neurons in the mouse motor cortex.

In this work, we use fluorescence micro-optical sectioning tomography system (fMOST)^13,19^ to acquire a whole mouse brain image data, which comes from a Thy-1 mouse line. Based on the fMOST dataset, we reconstruct pyramidal neuron morphology at single axon resolution with the cytoarchitecture information. By analyzing dendritic spreading patterns and axonal projection patterns, we identify two axonal projection patterns of PT neurons, and two dendritic spreading patterns of IT neurons in layer 5 of motor cortex, which may suggest the subtypes of pyramidal neurons, enrich the knowledge about the brain circuit connectivity the mouse brain motor cortex and provide evidence to explain the input-output function of single neuron^20–23^.

## Results

### The axon projection pattern at single axon resolution

**Figure 1:**
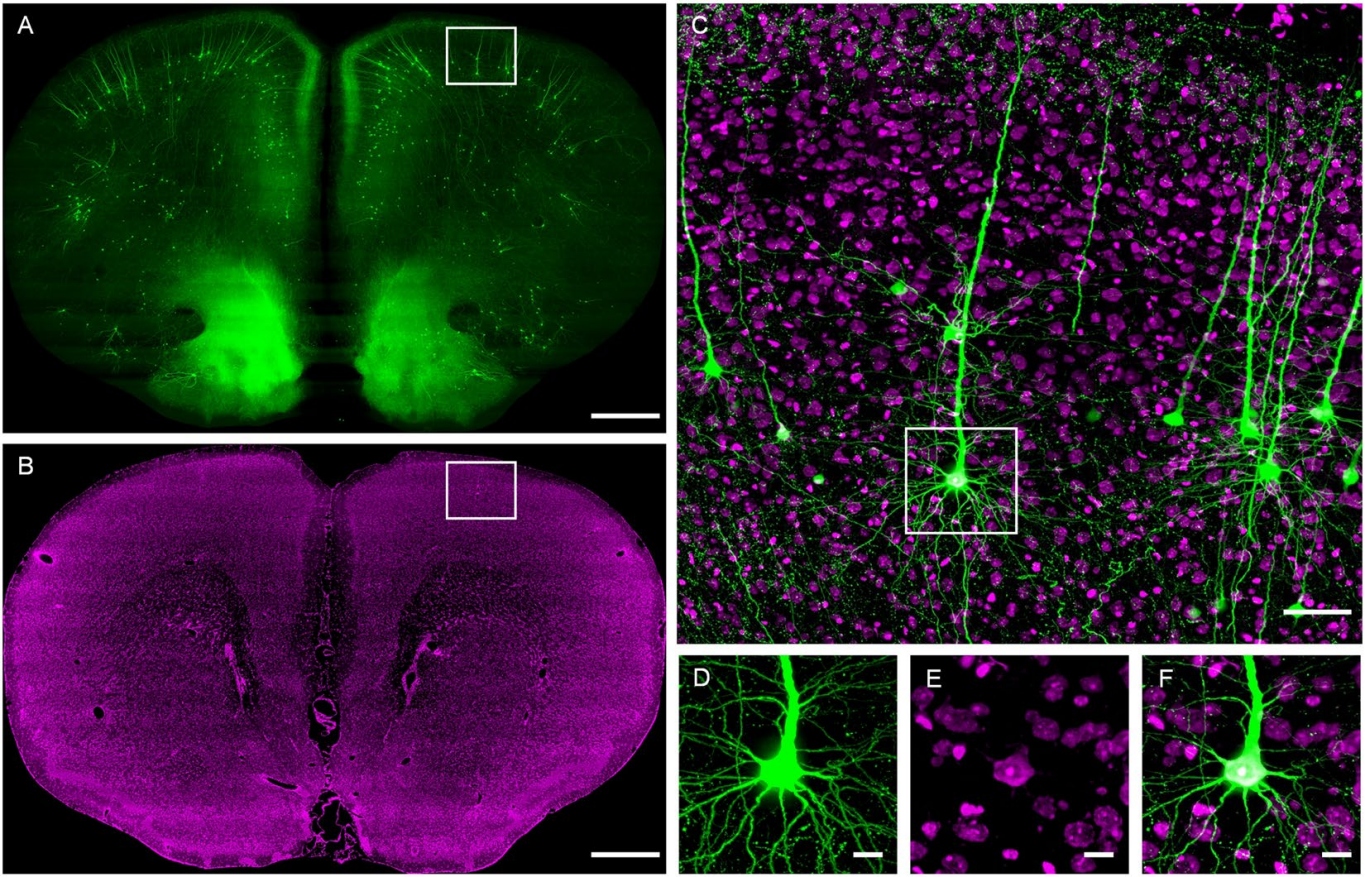
The GFP channel images and the PI channel images are aligned to each other. (**A**) A GFP coronal slice image from fMOST system for the neuron fiber tracing with 400 μm projection. (**B**) The corresponding PI image with 10 μm projection. (**C**) The overlapped image of the white boxes in A and B. (**D, E**) The raw signals of the white box area in C from the GFP and the PI channel respectively. (**F**) The overlapped image of D and E indicates the co-localization at single soma resolution. Scale bars: (**A**,**B**) 500 μm, (**C**) 50 μm, (**D**–**F**) 10 μm.

**Figure 2:**
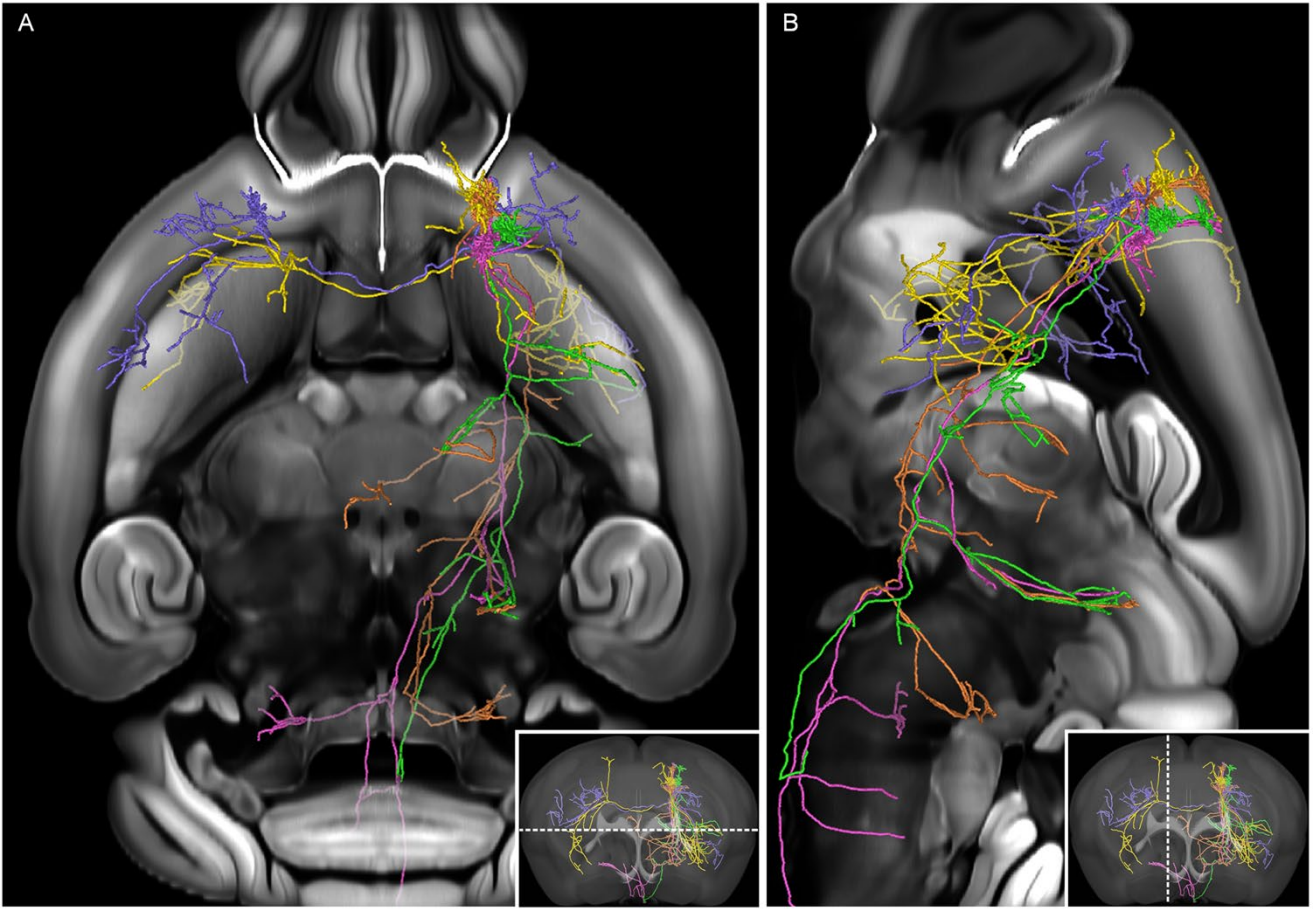
Five reconstructed pyramidal neurons with coded color across the whole mouse brain. (**A**) The neuron morphologies in the horizontal view whose position is shown by the white dash line in the coronal slice at the lower right corner. (**B**) The neuron morphologies in the sagittal view whose position is shown by the white dash line in the coronal slice at the lower right corner. The neurons are registered to the Allen Mouse Common Coordinate Framework version 3.

**Figure 3:**
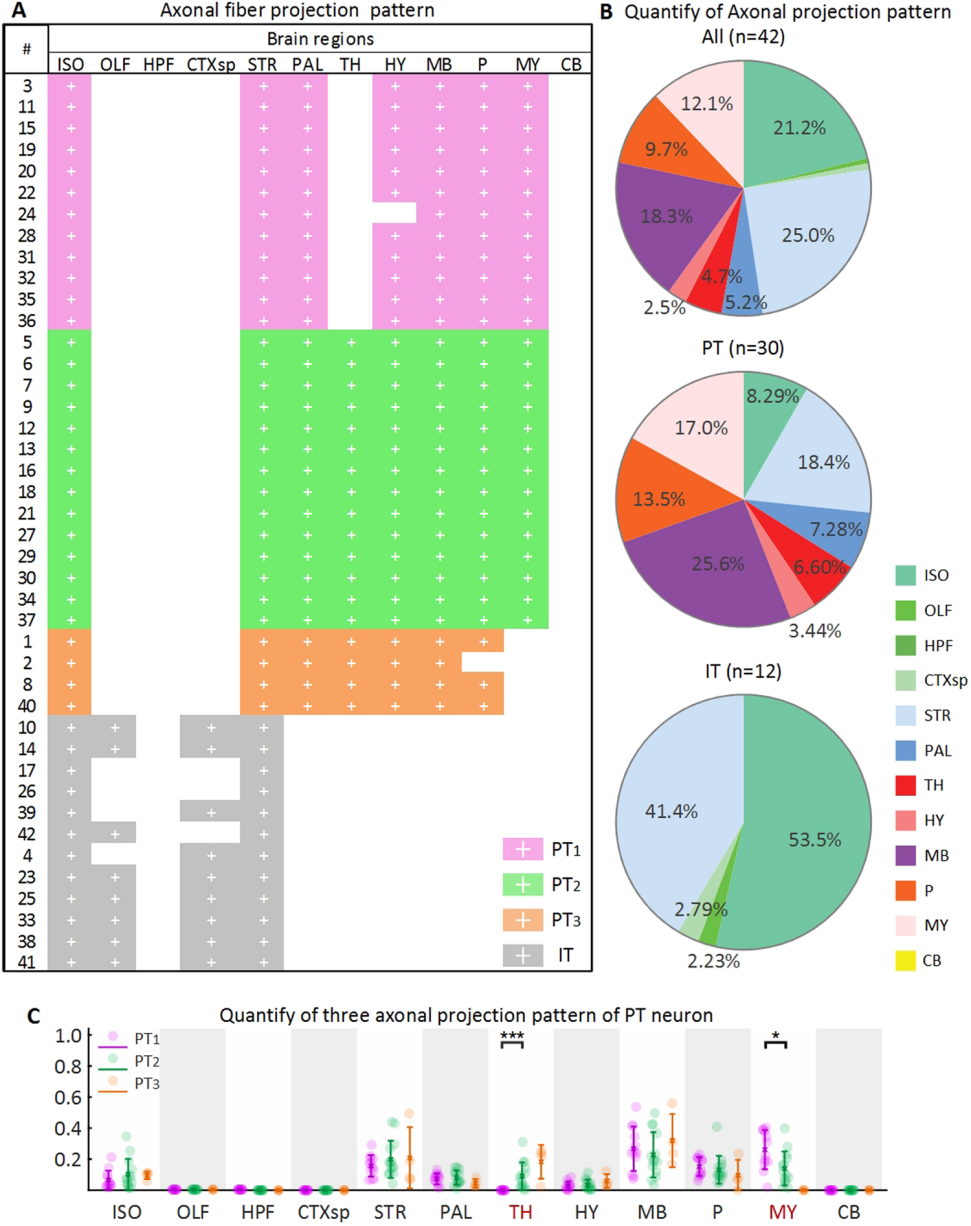
The axonal projection pattern statistics. (**A**) Axonal projection pattern of 42 neuron morphology colored based on 12 brain regions. 42 neurons are colored based on four different axonal projection patterns by grey (IT), pink (PT_1_), green (PT_2_), and orange (PT_3_). (**B**) Pie charts of axon projection ratio in the 12 brain regions. The neurons are classified as IT neurons and PT neurons by the contralateral or ipsilateral projection. (**C**) The relative fiber length ratio in brain regions of axonal projection patterns of PT neurons. Every dot represents the length ratio of a neuron, and a cross represents the average length ratio. The brain regions are defined in the Allen Mouse Common Coordinate Framework version 3. The used abbreviations are: ISO for Isocortex, OLF for Olfactory areas, HPF for Hippocampal formation, CTXsp for Cortical subplate, STR for Striatum, PAL for Pallidum, TH for Thalamus, HY for Hypothalamus, MB for Midbrain, P for Pons, MY for Medulla, and CB for Cerebellum. Confidence level is set to 0.05 (*p < 0.05, **p < 0.01, ***p < 0.0001).

From the whole brain imaging dataset at the single axon resolution (Fig. 1), we reconstruct 42 pyramidal neurons in layer 5 of the motor cortex of one mouse brain (Fig. 2). According to previous studies^1,7,24–27^, the axonal projection pattern can be a primary standard to assign the types to pyramidal neurons in the cortex. So we identify every brain region to which axons project to (Fig. 3A), and classify the 42 neurons to IT neurons (12 neurons) and PT neurons (30 neurons) according to their axonal projection patterns. From these projection patterns, all IT neurons have the contralateral projection, and target the isocortex (In this work, we use Allen Mouse Brain Connectivity Atlas^28^. In this brain atlas, the isocortex is a part of cerebral cortex, and contains many sub-regions such as somatomotor areas, somatosensory areas, visual areas. More information can be referred to Allen brain atlas.) and the striatum. On the other hand, all PT neurons have the ipsilateral projection, and target the isocortex, striatum, pallidum, and midbrain. Meanwhile, axons of all neurons project to the caudoputamen. Furthermore, we calculate the average percentage of 42 axon projection in 12 brain regions, which is a different distribution pie chart for IT and PT neurons’ (Fig. 3B). This is a summary of projection patterns of IT and PT neurons, although they exhibit different projection patterns.

Among 30 PT neurons, we identify two axonal projection patterns based on brain regions that PT axonal fibers project to, including PT_1_, PT_2_. The PT_1_ (12 neurons, colored with pink in Figs. 3A and 3C) neurons project to the medulla not to the thalamus. The PT_2_ (14 neurons, colored with green in Figs. 3A and 3C) neurons projection pattern is different from PT_1_ neurons that their projection destinations contain both the thalamus and the medulla. Also, we observe another projection pattern PT_3_ contains thalamus not medulla. However, the sample size of this pattern is too small (4 neurons), we do not analyze it further. The observation of PT_1_ and PT_3_ projection pattern is consistent with the work by Economo^7^.

### The dendrite spreading pattern partially relate to axon projection pattern

**Figure 4:**
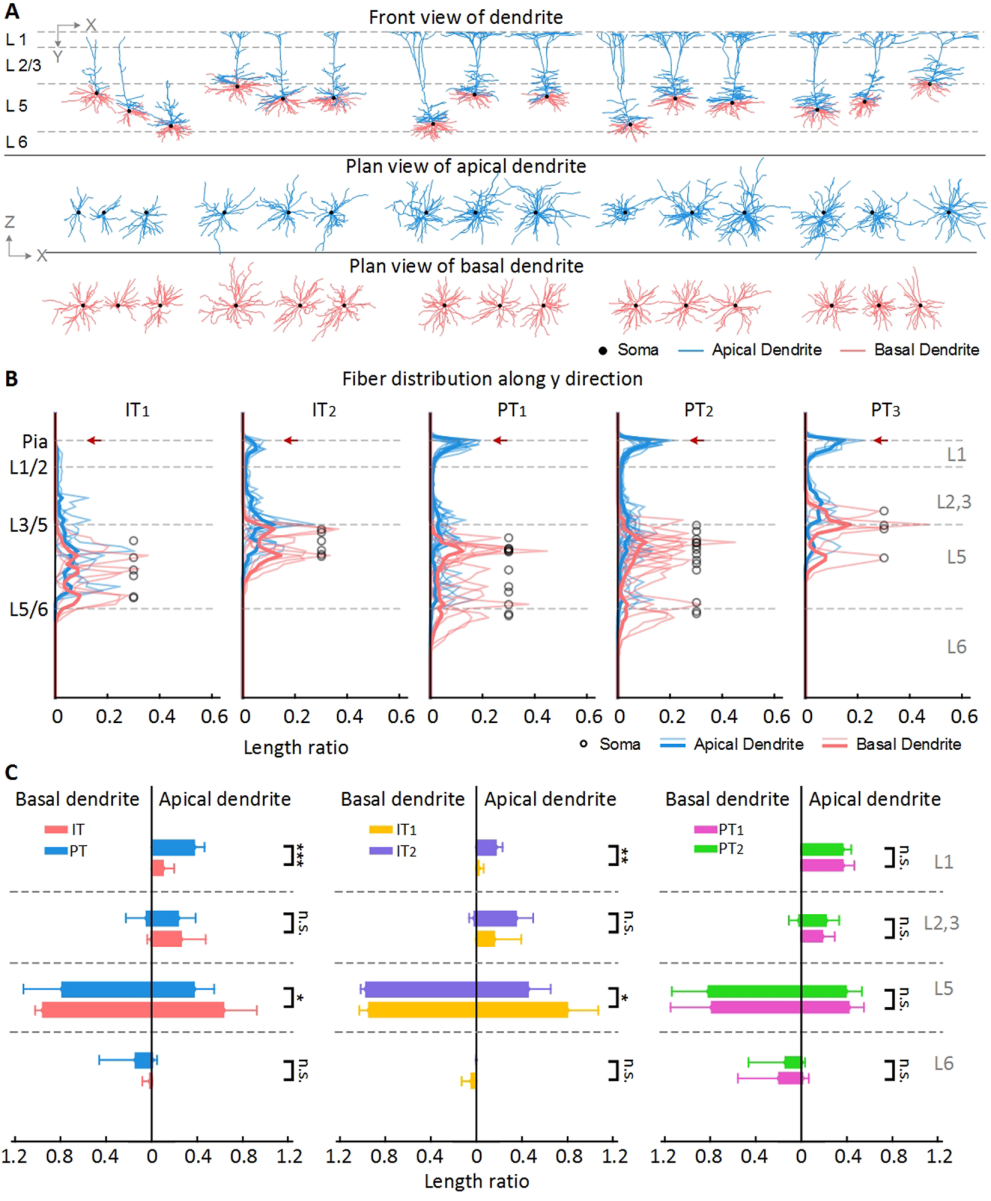
The dendritic spreading pattern of layer 5 pyramidal neurons in motor cortex. (**A**) The coronal and transverse view of 3D reconstructed dendrite morphology. (**B**) The apical (carnation) and basal (blue) dendrite spreading curves along y direction in (**A**). The thin lines are for the single neuron spreading curves and the bold lines are for the average spreading curves of each type. (**C**) The apical and basal fiber length ratio in different layers. Squared brackets on the right side of the plot indicate the pair of statistics with significant difference for apical dendritic fibers. For mean ± standard deviation of the values plotted in this figure, see supplementary material table 2. The grey dash lines are layer borders. Confidence level is set to 0.05 (*p* value). Confidence level is set to 0.05 (*p < 0.05, **p < 0.01, ***p < 0.0001).

For IT and PT neurons, it is widely known that differences exist in their apical dendrites morphology^29^. IT neurons have slender apical dendrite tufts in the superficial layer, while PT neurons have thick tufts^4,25^. This difference can be clearly observed (Fig. 4A) from our reconstructed neurons. We plot the fiber spreading curves in the vertical direction of the neocortex (Fig. 4B), and find that the PT neurons have more apical dendritic fibers in layer 1 more than IT neurons (Fig. 4C; Wilcoxon rank sum test, *p* = 5.78×10^−7^). While relative length of apical dendritic fibers from IT is longer than PT neurons in layer 5 (Wilcoxon rank sum test, *p* = 0.020).

The dendritic morphology of IT neurons shows two different spreading patterns. Specifically, IT_1_ pattern have less apical dendritic fibers appearing in layer 1 than IT_2_ pattern (Fig. 4C; Wilcoxon rank sum test, *p* = 0.002). IT_2_ apical dendrites have similar spreading pattern to PT neurons along the vertical direction of the neocortex. They not only reach the pia but also form a tiny tuft as shown in Fig. 4B. However, apical dendritic fibers of IT_2_ pattern in layer 1 do not have as much percentage as PT apical dendritic fibers. In the case of basal dendrites, although the dendrite fibers always spread around their somata in the vertical direction of the cortex, there is no significant difference in the spatial spreading pattern of dendritic fibers among neurons have PT_1_ or PT_2_ axonal projection pattern. Besides, the basal dendritic fibers have a large percentage in layer 5, as shown in Fig. 4C, because the somata reside in layer 5 and basal dendritic fibers are usually distributed around the somata. These three dendritic spreading patterns are morphologically similar with the neurons in layer 5 of mouse barrel cortex ported by Larsen^30^. After all, difference in apical and basal dendritic fiber distribution among neurons may not be a coincidence since these neurons may not perform the same function^31^.

### The spatial distribution of soma

**Figure 5:**
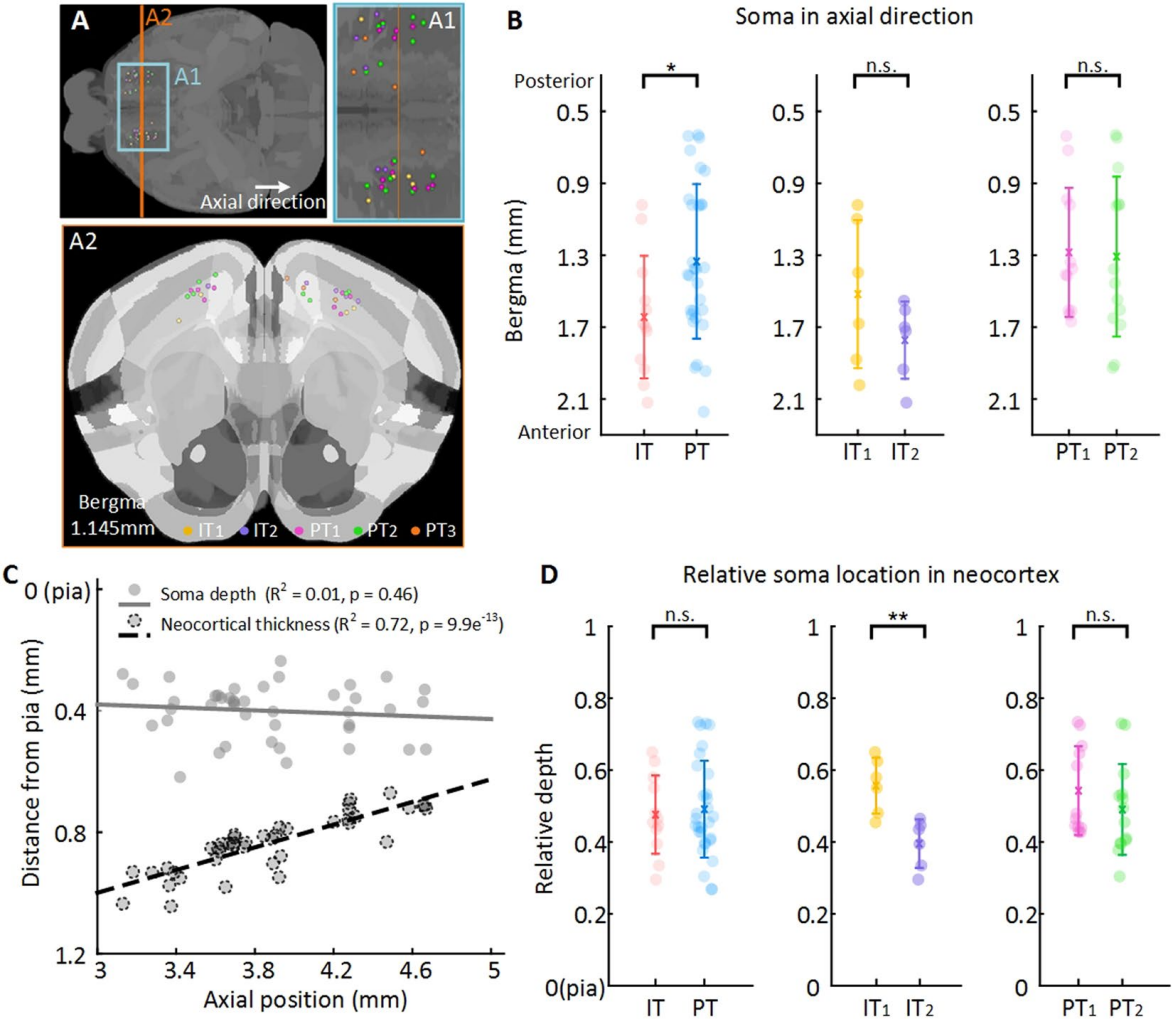
The soma spatial distribution of layer 5 pyramidal neurons in motor cortex. (**A**) The horizontal view of the soma location in whole brain. (A1) Enlarged area of the blue block in A. (A2) the coronal view of the soma location indicated in A with orange line. (**B**) The scatter plot of absolute somata location in axial direction. The axial positions of somata are in consist with the Allen Mouse Common Coordinate Framework version 3. (**C**) Linear regression of the soma depth, neocortical thickness and the axial position. (**D**) The scatter plot of relative somata location in neocortex. Soma is colored as the same with previous figures based on their morphology pattern. Confidence level is set to 0.05 (*p < 0.05, **p < 0.01, ***p < 0.0001).

The somata of 42 neurons are distributed in the primary and the secondary motor cortexes. The spatial distributions of somata for each fiber distribution pattern of pyramidal neurons are different from each other. Firstly, we analyze the soma position in axial direction. The IT somata stay relatively anterior to PT somata (Fig. 5B; Wilcoxon rank sum test, *p* = 0.020). While, this significant difference does not exist between neurons that show different axonal projection pattern or dendritic spreading pattern. Then, we measure the relative distance form soma to pia to analyze relationship between the soma laminar location and morphological patterns. From the analysis (Fig. 5D), the IT_2_ somata stay significantly deeper than IT_1_ somata in neocortex. This situation has also been observed in somatosensory cortex^32^. While this difference does not exist between IT neurons and PT neurons, or neurons with PT_1_ axonal projection pattern and PT_2_ axonal projection pattern.

Also, we analyze the relation between distance from soma to pia (soma depth), the neocortical thickness and soma position in axial direction. From the linear regression result (Fig. 5C), the neocortical thickness has a medium linear correlation with axial direction, while soma depth does not have significant correlation with axial direction. This linear regression result suggests that the somata we pick to reconstructed may not stay at the same relative depth of the neocortex.

## Discussion

The neuron cell type has been a complex and fundamental scientific question since Cajal published his research^33^ a hundred years ago. From then on, the neuron anatomy has become one of the primary criteria of cell typing^2,29,34,35^. However, for pyramidal neurons, whose axon can project to brain region across the whole mouse brain, it is difficult to obtain sufficient number of axon samples because it is difficult to acquire whole brain images at the single axon resolution. Previous studies have been restricted to the local areas and seldom reached the subcortical areas at the single axon resolution. The fMOST system^13,36,37^ and Janelia team^17^ confront this challenge by developing a micro-optical imaging pipeline specifically for the whole brain imaging. The fMOST system provides both a whole brain imaging dataset at single axon resolution and the cytoarchitectonic information. From an fMOST dataset of a Thy-1 mouse brain, we reconstruct the neuronal fibers of the pyramidal neurons whose somata are in the layer 5 of the motor cortex. We categorize these samples into four morphological patterns based on their axonal projection pattern, dendritic spreading pattern. There are two axonal projection patterns for PT neurons and two dendritic spreading patterns for IT neurons. The patterns of PT neurons are all having multiple projection targets in subcortical areas. These targets have been reported in previous work^7,38^. In our work, PT_1_ axonal fibers project to medulla but not to thalamus, and PT_2_ neurons project both to thalamus and medulla. PT_1_ project patterns are consistent with Economo’s work^7^. Interestingly, these exclusive projections to either medulla or thalamus have also been reported in the work about PT neurons in somatosensory cortex from Rojas-Piloni^38^. And Rojas also report that some of the PT neurons project both to thalamus and medulla. The PT projection patterns are also reported for visual cortex, as seen in^39^. However, we do not find the non-striatal projection pattern, which is reported by Kim^39^, among our reconstructed neurons. We suspect this is related to the different function of the motor cortex and the visual cortex.

We analyze the pyramidal neuron morphology according to the spatial information, specifically, brain regions and nucleus. According to Peters’ rule^40^, “circuit connectivity among neuron types is statistically determined based on the regional colocation of the potential presynaptic axons and postsynaptic dendrites”, we suspect that the PT_1_ and PT_2_ have the similar information input because of the similar dendritic pattern, but modular downstream neurons with different time course, which may influence some mouse actions, like the functions of two PT subtype neurons reported by Economo *et al*.^7^. Furthermore, hypothetically, considering the plasticity of the neuron morphology in motor cortex are influenced by the function of learning^41^, PT_2_ axonal projection patterns which seems a combination pattern of PT_1_ and PT_3_ might change to PT_1_ or PT_3_ pattern after some learning training.

In conclusion, we analyze the morphological property of 42 neurons at the layer 5 of mouse motor cortex to identify two axonal projection patterns of PT neuron, and two dendritic spreading patterns of IT neurons. These morphological patterns may suggest subtypes of pyramidal neurons in layer 5 of motor cortex. The dataset used in this work is a contribution to the community, which can provide more evidence to the known observation and lay the foundation for future discovery.

## Methods

### Tissue preparation

We select an adult male Thy1 eYFP H-line transgenic mouse^42^ as the imaging target^43,44^. The mouse is anesthetized using the 1% solution of sodium pentobarbital. Then, 0.01 M PBS (Sigma-Aldrich Inc., St. Louis, MO, USA) is used for intracardial perfusion, followed by 4% paraformaldehyde (Sigma-Aldrich Inc., St. Louis, MO, USA) and 2.5% sucrose in 0.01 M PBS. We fix the mouse brain in 4% paraformaldehyde at 4 °C for 24 hours. The mouse brain is subsequently rinsed overnight at 4 °C in a 0.01 M PBS solution that contains 2.5% sucrose. Then we dehydrate the brain via immersion in a graded series of ethanol mixtures: 50% ethanol, 75% ethanol, 100% ethanol. After that, we replace the ethanol with graded series of xylene (with pure ethanol): 50% xylene and 100% xylene. Finally, we impregnate with the Lowicryl HM20 resin embedding (inxylene): 50% HM20, 75% HM20, 100% HM20 and 100% HM20. All dehydration and infiltration procedures are performed at 4 °C. All animal experiments are performed according to the procedures approved by the Institutional Animal Ethics Committee of Huazhong University of Science and Technology.

### Whole-brain high resolution imaging with propidium staining (PI)

The mouse brain is sectioned and imaged automatically using the fMOST system^13,19^ Prior to the imaging process, the resin-embedded whole mouse brain sample is fixed by a customized clamp in the anterior-posterior direction in a water bath on a 3D translation stage. The sample is immersed in a water bath filled with the PI-Na_2_CO_3_ solution. Sectioning is achieved using a fixed diamond knife and a 3D translation stage. The x-axis of the translation stage is the sectioning direction. We image the sample surface and section a slice off the surface, then image lower surface. As a result, the serial slices are natural spliced. The single sectioned physical slice thickness is set to 1 μm. The fMOST system automatically acquires a brain-wide dataset with the stripe imaging process. The GFP channel and the PI channel signal are recorded at a voxel size of 0.2 × 0.2 × 1 μm^3^ simultaneously. The size of the obtained image dataset exceeds 51.7 terabytes for one sample including both the GFP and the PI channel.

### Image preprocessing

We preprocess the imaging dataset with the GFP channel and the PI channel according to the published protocol^13^. We use a set of automatic procedures to improve the image quality and facilitate further processing and analysis^45^. Specifically, we stitch the acquired stripes, align the PI channel and the GFP channel images, and correct the illumination of images. The GFP channel is saved using a 16-bit depth LZW compression TIFF format. The PI channel images are saved at an 8-bit depth.

### Single neuron reconstruction

We manually reconstruct 42 neurons based on the whole brain fluorescence imaging dataset at 0.2 × 0.2 × 1 μm^3^ voxel resolution using Amira software (v6.1.1, FEI, France) with the TDat data accessing module^46^. The brain imaging dataset used for the single neuron reconstruction contains more than ten thousand coronal slices at 1 μm axial resolution. The fibers can be separated clearly by their brightness and thickness at this resolution. We choose some somata with high fluorescent signals in motor cortex as the starting point for the whole neuron reconstruction. The neuron tracing process is similar to the approaches used by other researchers^13,18,37,47^. The annotators have been trained for neuron reconstruction for 6 months. Moreover, back-to-back verifications are performed for each reconstructed neuron.

To check the validity of the reconstructed neuron structure, we transform the file in the SWC format saved by Amira to the ASC format file using Neuronland software (Neuromorpho.org). In Neurolucida 360 software (Neurolucida 360, version 2.70.1, MBF Bioscience, Williston, VT), we subsequently label the fibers as axon, apical dendrite or basal dendrite according to the manual reconstruction result.

### Brain regions segmentation and nuclei recognition

The images of propidium staining (PI) channel are obtained at the same time as the fluorescence channel using the fMOST whole brain imaging system. The PI channel image can provide cytoarchitecture information, which is necessary for nuclei segmentation. The dataset for one whole mouse brain obtained by the fMOST system can contain over ten thousand consecutive PI stained images. These high-resolution PI channel images can be used to allocate the neuronal cell body and delineate the brain regions and nuclei.

The cortex layer should be annotated to understand the neuron morphology. We manually label the layer borders of the cortex for the whole brain maximum intensity projection images according to the somata size. The maximum intensity projection images are obtained based the cytoarchitecture imaging dataset at 0.2 × 0.2 × 1 μm^3^ voxel resolution through a depth of 10 μm projection in the coronal plane using the Amira software (v6.1.1, FEI, France). To register the dendrites in the different cortex areas to the same template for comparison and analysis, we label the main branch of the apical dendrite using the same imaging dataset but with an intensity projection depth of 3 μm in the coronal plane. The similar process has been used in the literature previously^37^. For the somata allocation, we use the layer borders that have been drawn for dendrites.

For axons projection pattern analysis, the whole brain is semi-automatically segmented into 12 brain regions according to the Allen Reference Atlas^48^. The original imaging dataset is down sampled into 10 × 10 × 10 μm^3^ voxel resolution, and registered to the template of the Allen Mouse Common Coordinate Framework version 3 (CCF v3) using the affine transformation and a symmetric image normalization^49–51^ in Advanced Normalization Tools (ANTS)^50,52^. Then three experienced researchers manually validate and amend the brain regions back-to-back according to the PI images in Amira software (v6.1.1, FEI, France).

### The analysis on axonal and dendritic fiber patterns

We locate the brain regions that axonal fibers project to, and classify the 42 neurons morphologies into IT (n = 12) and PT (n = 30) projection pattern. Among PT projection patterns, based on the axonal fibers in thalamus and medulla, we classify the PT projection pattern into PT_1_ (n = 12), PT_2_ (n = 14) and PT_3_ (n = 4) projection pattern. Because the small sample size of PT_3_, we do not analyze it further.

For dendrites, we first rotate the neuron and layer borders according to the direction of the main branch in apical dendrite using principle component analysis (PCA). We subsequently align dendrites to the vertical direction of cortical layer boundary. Based on this template, we plot the dendrite spreading curves along the vertical axis with the laminar arbitrary units (AUs). The AU size is defined such that different neurons in the same layer will have the same AU value. As a consequence, the absolute size of the AUs for every neuron is different while the relative layer length of AUs for each neuron is the same. Similar treatment has been presented in a previous paper^37^.

### Statistical analysis

Statistical significances are analyzed using MATLAB (version 2016a, The MathWorks, Natick, MA) and statistical comparisons use two-side Wilcoxon rank sum test. All measurements are listed as the mean ± standard deviation.

## Supplementary information

Supplementary materials.

## Data Availability

The neuron morphologies and codes used in this study are available from NeuroMorpho.Org (http://www.neuromorpho.org/NeuroMorpho_Linkout.jsp?PMID=32405029).
