# Author Correction: Anatomically revealed morphological patterns of pyramidal neurons in layer 5 of the motor cortex

**DOI:** 10.1038/s41598-022-08599-x

**Published:** 2022-03-15

**Authors:** Siqi Jiang, Yue Guan, Shangbin Chen, Xueyan Jia, Hong Ni, Yalun Zhang, Yutong Han, Xue Peng, Can Zhou, Anan Li, Qingming Luo, Hui Gong

**Affiliations:** 1 Britton Chance Center for Biomedical Photonics, Key Laboratory for Biomedical Photonics of Ministry of Education, Wuhan National Laboratory for Optoelectronics-Huazhong University of Science and Technology, Wuhan, 430074 China; 2 HUST-Suzhou Institute for Brainsmatics, JITRI Institute for Brainsmatics, Suzhou, 215100 China

Correction to: *Scientific Reports* 10.1038/s41598-020-64665-2, published online 13 May 2020

In the original paper, the Data Availability statement was incomplete.

“The neuron morphologies and codes used in this study are available from the corresponding author upon request”

now reads:

“The neuron morphologies and codes used in this study are available from NeuroMorpho.Org (http://www.neuromorpho.org/NeuroMorpho_Linkout.jsp?PMID=32405029)”

The original Article has been corrected.

