# Supplementary material for: Anatomically revealed morphological patterns of pyramidal neurons in layer 5 of the motor cortex
Source: Sci Rep. 2020 May 13;10:7916. doi: 10.1038/s41598-020-64665-2 (PMC7220918; doi:10.1038/s41598-020-64665-2)
Supplement: Supplementary file 1 — Supplementary materials. [file 41598_2020_64665_MOESM1_ESM.docx]

Title: Anatomically revealed morphological patterns of pyramidal neurons in layer 5 of the motor cortex

Siqi Jiang^1^, Yue Guan^1^, Shangbin Chen^1^, Xueyan Jia^2^, Hong Ni^1^, Yalun Zhang^1^, Yutong Han^1^, Xue Peng^1^, Can Zhou^1^, Anan Li^1, 2^, Qingming Luo^1, 2^, & Hui Gong^1, 2^*

^1^Britton Chance Center for Biomedical Photonics, Key Laboratory for Biomedical Photonics of Ministry of Education, Wuhan National Laboratory for Optoelectronics-Huazhong University of Science and Technology, Wuhan 430074, China.

^2^HUST-Suzhou Institute for Brainsmatics, JITRI Institute for Brainsmatics, Suzhou 215100, China.

Corresponding author: Hui Gong

Email: [huigong@mail.hust.edu.cn](mailto:huigong@mail.hust.edu.cn)

| **#** | **Absolute axonal fiber length in brain regions (μm)** | | | | | | | | | | | |
| --- | --- | --- | --- | --- | --- | --- | --- | --- | --- | --- | --- | --- |
|  | **ISO** | **OLF** | **HPF** | **CTXsp** | **STR** | **PAL** | **TH** | **HY** | **MB** | **P** | **MY** | **CB** |
| 3 | 849.4 | 0.0 | 0 | 0.0 | 2570.4 | 2418.4 | 0.0 | 325.8 | 9367.3 | 8134.5 | 15059.0 | 0 |
| 11 | 359.1 | 0.0 | 0 | 0.0 | 2640.0 | 1266.4 | 0.0 | 324.5 | 1518.2 | 3148.5 | 5974.5 | 0 |
| 15 | 2682.5 | 0.0 | 0 | 0.0 | 5636.3 | 2335.5 | 0.0 | 171.6 | 11391.3 | 1651.7 | 3331.8 | 0 |
| 19 | 1066.7 | 0.0 | 0 | 0.0 | 1697.7 | 729.9 | 0.0 | 890.0 | 5478.0 | 1101.6 | 2343.9 | 0 |
| 20 | 165.0 | 0.0 | 0 | 0.0 | 2521.9 | 1214.0 | 0.0 | 14.5 | 839.6 | 2346.8 | 4368.4 | 0 |
| 22 | 1640.9 | 0.0 | 0 | 0.0 | 2029.6 | 1542.9 | 0.0 | 571.7 | 1033.4 | 2737.3 | 2283.2 | 0 |
| 24 | 441.4 | 0.0 | 0 | 0.0 | 3529.7 | 1172.5 | 0.0 | 0.0 | 3030.4 | 1188.3 | 2711.7 | 0 |
| 28 | 1081.3 | 0.0 | 0 | 0.0 | 1620.8 | 382.8 | 0.0 | 413.3 | 8045.2 | 2335.3 | 9387.2 | 0 |
| 31 | 153.4 | 0.0 | 0 | 0.0 | 1579.4 | 478.8 | 0.0 | 364.6 | 3108.0 | 1443.4 | 3749.0 | 0 |
| 32 | 454.8 | 0.0 | 0 | 0.0 | 2822.8 | 1301.4 | 0.0 | 529.8 | 2950.1 | 1486.6 | 4265.7 | 0 |
| 35 | 419.2 | 0.0 | 0 | 0.0 | 1790.8 | 1094.9 | 0.0 | 263.3 | 7515.6 | 2692.0 | 242.5 | 0 |
| 36 | 5024.7 | 0.0 | 0 | 0.0 | 1774.6 | 397.8 | 0.0 | 2033.8 | 5776.6 | 4754.4 | 4394.2 | 0 |
| 5 | 1788.8 | 0.0 | 0 | 0.0 | 2648.8 | 718.7 | 2038.8 | 1938.3 | 5600.9 | 10457.3 | 427.6 | 0 |
| 6 | 4974.3 | 0.0 | 0 | 0.0 | 2568.4 | 1694.4 | 353.9 | 219.0 | 943.7 | 1445.7 | 2303.2 | 0 |
| 7 | 947.0 | 0.0 | 0 | 0.0 | 2618.6 | 3568.7 | 2316.2 | 441.1 | 10216.7 | 1115.2 | 2949.2 | 0 |
| 9 | 987.0 | 0.0 | 0 | 0.0 | 3772.7 | 1519.8 | 235.4 | 931.0 | 2183.1 | 2071.1 | 2346.7 | 0 |
| 12 | 915.9 | 0.0 | 0 | 0.0 | 5230.3 | 2338.3 | 1673.8 | 1570.4 | 12079.5 | 2516.7 | 2044.5 | 0 |
| 13 | 160.0 | 0.0 | 0 | 0.0 | 1755.3 | 103.3 | 427.2 | 1383.3 | 2241.7 | 1255.7 | 4845.0 | 0 |
| 16 | 2889.8 | 0.0 | 0 | 0.0 | 2865.6 | 2308.2 | 4855.0 | 2.0 | 540.2 | 1448.1 | 706.4 | 0 |
| 18 | 330.0 | 0.0 | 0 | 0.0 | 16405.1 | 1699.8 | 6672.8 | 599.5 | 7268.1 | 1896.9 | 2408.2 | 0 |
| 21 | 2524.7 | 0.0 | 0 | 0.0 | 3741.6 | 1270.4 | 84.7 | 261.8 | 12915.5 | 2538.8 | 2602.6 | 0 |
| 27 | 10478.2 | 0.0 | 0 | 0.0 | 1733.3 | 2359.8 | 1069.7 | 705.4 | 9805.4 | 6202.8 | 9691.6 | 0 |
| 29 | 1181.6 | 0.0 | 0 | 0.0 | 1608.4 | 1559.7 | 2815.6 | 739.6 | 1309.6 | 2928.4 | 3144.3 | 0 |
| 30 | 131.9 | 0.0 | 0 | 0.0 | 1616.1 | 1494.6 | 1173.3 | 289.1 | 1289.0 | 1777.9 | 3016.3 | 0 |
| 34 | 1156.5 | 0.0 | 0 | 0.0 | 7146.5 | 1215.9 | 3443.9 | 13.4 | 8851.5 | 2086.4 | 325.8 | 0 |
| 37 | 2688.7 | 0.0 | 0 | 0.0 | 9002.4 | 1162.9 | 120.0 | 110.3 | 3835.6 | 2294.7 | 1692.6 | 0 |
| 1 | 1272.7 | 0.0 | 0 | 0.0 | 6484.9 | 385.1 | 275.8 | 1599.2 | 2206.5 | 911.7 | 0.0 | 0 |
| 2 | 1074.0 | 0.0 | 0 | 0.0 | 1531.4 | 347.6 | 4632.5 | 920.9 | 10842.8 | 0.0 | 0.0 | 0 |
| 8 | 1508.1 | 0.0 | 0 | 0.0 | 2654.7 | 666.7 | 3543.6 | 269.6 | 4376.2 | 1089.0 | 0.0 | 0 |
| 40 | 3369.2 | 0.0 | 0 | 0.0 | 2261.3 | 2608.6 | 6978.2 | 1545.5 | 7613.3 | 7538.8 | 0.0 | 0 |
| 10 | 28329.9 | 54.3 | 0 | 1891.7 | 3133.0 | 0.0 | 0.0 | 0.0 | 0.0 | 0.0 | 0.0 | 0 |
| 14 | 14021.3 | 3341.6 | 0 | 6.8 | 402.8 | 0.0 | 0.0 | 0.0 | 0.0 | 0.0 | 0.0 | 0 |
| 17 | 9317.2 | 0.0 | 0 | 0.0 | 7.2 | 0.0 | 0.0 | 0.0 | 0.0 | 0.0 | 0.0 | 0 |
| 26 | 7160.4 | 0.0 | 0 | 0.0 | 1428.6 | 0.0 | 0.0 | 0.0 | 0.0 | 0.0 | 0.0 | 0 |
| 39 | 19218.7 | 0.0 | 0 | 210.0 | 7742.3 | 0.0 | 0.0 | 0.0 | 0.0 | 0.0 | 0.0 | 0 |
| 42 | 3231.9 | 6.0 | 0 | 0.0 | 6691.6 | 0.0 | 0.0 | 0.0 | 0.0 | 0.0 | 0.0 | 0 |
| 4 | 5007.7 | 0.0 | 0 | 844.8 | 13846.4 | 0.0 | 0.0 | 0.0 | 0.0 | 0.0 | 0.0 | 0 |
| 23 | 10623.6 | 64.7 | 0 | 304.7 | 23785.3 | 0.0 | 0.0 | 0.0 | 0.0 | 0.0 | 0.0 | 0 |
| 25 | 9804.4 | 294.2 | 0 | 570.6 | 26011.3 | 0.0 | 0.0 | 0.0 | 0.0 | 0.0 | 0.0 | 0 |
| 33 | 19670.0 | 692.8 | 0 | 6363.0 | 30295.5 | 0.0 | 0.0 | 0.0 | 0.0 | 0.0 | 0.0 | 0 |
| 38 | 10785.2 | 1086.7 | 0 | 1628.3 | 11357.9 | 0.0 | 0.0 | 0.0 | 0.0 | 0.0 | 0.0 | 0 |
| 41 | 12964.5 | 457.9 | 0 | 1056.7 | 26514.6 | 0.0 | 0.0 | 0.0 | 0.0 | 0.0 | 0.0 | 0 |

**Supplementary material table S1** The absolute length of axonal fiber in 12 brain regions. PT_1_ axonal projection pattern contains medulla not thalamus, PT_2_ axonal projection pattern contains both thalamus and medulla, PT_3_ axonal projection pattern contains thalamus not medulla. This three projection pattern are colored with pink (PT_1_), green (PT_2_) and orange (PT_3_). The used abbreviations are: ISO for Isocortex, OLF for Olfactory areas, HPF for Hippocampal formation, CTXsp for Cortical subplate, STR for Striatum, PAL for Pallidum, TH for Thalamus, HY for Hypothalamus, MB for Midbrain, P for Pons, MY for Medulla, CB for Cerebellum, and FT for fiber tracks. In the axonal projection analysis, we do not take the FT into account, because the axon in FT seldom make connection with other neurons.

| **#** | **Basal dendrite (μm)** | | | | | **Apical dendrite (μm)** | | | | |
| --- | --- | --- | --- | --- | --- | --- | --- | --- | --- | --- |
|  | **layer 1** | **layer 2/3** | **layer 5** | **layer 6** | **total length** | **layer 1** | **layer 2/3** | **layer 5** | **layer 6** | **total length** |
| 3 | 0.0 | 0.0 | 4172.9 | 0.0 | 4172.9 | 1950.1 | 436.7 | 1225.0 | 0.0 | 3611.7 |
| 11 | 0.0 | 0.0 | 3311.4 | 0.0 | 3311.4 | 2195.0 | 2403.1 | 1452.6 | 0.0 | 6050.8 |
| 15 | 0.0 | 0.0 | 3643.2 | 0.0 | 3643.2 | 2591.4 | 2038.0 | 1885.5 | 0.0 | 6514.9 |
| 19 | 0.0 | 0.0 | 2824.4 | 642.7 | 3467.1 | 1741.5 | 488.7 | 1150.4 | 0.0 | 3380.6 |
| 20 | 0.0 | 0.0 | 399.8 | 3979.3 | 4379.2 | 1412.1 | 706.6 | 3397.7 | 83.5 | 5600.0 |
| 22 | 0.0 | 0.0 | 2952.5 | 0.0 | 2952.5 | 2059.7 | 1311.9 | 1969.6 | 0.0 | 5341.2 |
| 24 | 0.0 | 0.0 | 2145.3 | 0.0 | 2145.3 | 1691.9 | 1379.2 | 1661.4 | 0.0 | 4732.5 |
| 28 | 0.0 | 0.0 | 4375.1 | 202.8 | 4577.9 | 1912.7 | 276.8 | 3213.2 | 0.0 | 5402.7 |
| 31 | 0.0 | 0.0 | 232.2 | 2763.0 | 2995.2 | 1055.0 | 574.2 | 2151.6 | 777.8 | 4558.7 |
| 32 | 0.0 | 0.0 | 3063.1 | 0.0 | 3063.1 | 2655.6 | 1148.7 | 2423.8 | 0.0 | 6228.1 |
| 35 | 0.0 | 0.0 | 3163.9 | 0.0 | 3163.9 | 2024.4 | 1072.8 | 2847.6 | 0.0 | 5944.8 |
| 36 | 0.0 | 0.0 | 2115.5 | 1401.6 | 3517.1 | 1583.1 | 615.9 | 3298.1 | 0.0 | 5497.1 |
| 5 | 0.0 | 0.0 | 3612.0 | 0.0 | 3612.0 | 2298.0 | 972.6 | 1784.1 | 0.0 | 5054.7 |
| 6 | 0.0 | 0.0 | 1816.2 | 999.0 | 2815.2 | 1676.3 | 357.2 | 2645.1 | 0.0 | 4678.7 |
| 7 | 0.0 | 208.7 | 3247.7 | 0.0 | 3456.4 | 2893.0 | 2582.8 | 1260.4 | 0.0 | 6736.3 |
| 9 | 0.0 | 0.0 | 3457.8 | 0.0 | 3457.8 | 2949.8 | 1463.1 | 2459.4 | 0.0 | 6872.3 |
| 12 | 0.0 | 1011.5 | 2286.5 | 0.0 | 3298.0 | 2584.9 | 3246.5 | 844.8 | 0.0 | 6676.1 |
| 13 | 0.0 | 0.0 | 759.3 | 2469.8 | 3229.1 | 1115.1 | 632.6 | 2250.2 | 283.6 | 4281.4 |
| 16 | 0.0 | 0.0 | 2143.7 | 0.0 | 2143.7 | 1667.2 | 1461.5 | 2918.5 | 0.0 | 6047.1 |
| 18 | 0.0 | 0.0 | 3880.7 | 0.0 | 3880.7 | 1481.9 | 1099.9 | 2694.8 | 0.0 | 5276.5 |
| 21 | 0.0 | 0.0 | 3943.1 | 0.0 | 3943.1 | 2604.4 | 989.2 | 3211.6 | 0.0 | 6805.2 |
| 27 | 0.0 | 0.0 | 3669.3 | 13.4 | 3682.7 | 3107.9 | 918.9 | 4002.2 | 0.0 | 8029.0 |
| 29 | 0.0 | 111.9 | 4499.2 | 0.0 | 4611.1 | 2748.6 | 2057.1 | 1930.4 | 0.0 | 6736.1 |
| 30 | 0.0 | 0.0 | 198.9 | 3487.2 | 3686.1 | 1192.1 | 731.0 | 2071.7 | 239.9 | 4234.7 |
| 34 | 0.0 | 13.0 | 3925.5 | 0.0 | 3938.5 | 2590.8 | 1399.4 | 2300.1 | 0.0 | 6290.3 |
| 37 | 0.0 | 0.0 | 3655.7 | 0.0 | 3655.7 | 3178.6 | 1599.1 | 2675.5 | 0.0 | 7453.1 |
| 1 | 0.0 | 976.9 | 1948.7 | 0.0 | 2925.6 | 3112.4 | 3155.8 | 219.1 | 0.0 | 6487.3 |
| 2 | 0.0 | 3050.6 | 484.8 | 0.0 | 3535.3 | 3548.8 | 3052.8 | 0.0 | 0.0 | 6601.6 |
| 8 | 0.0 | 199.6 | 2824.4 | 0.0 | 3023.9 | 1504.2 | 3473.5 | 275.0 | 0.0 | 5252.6 |
| 40 | 0.0 | 0.0 | 3710.3 | 0.0 | 3710.3 | 2586.7 | 444.5 | 3564.5 | 0.0 | 6595.7 |
| 10 | 0.0 | 0.0 | 1895.2 | 391.5 | 2286.7 | 0.0 | 0.0 | 2233.6 | 0.0 | 2233.6 |
| 14 | 0.0 | 11.9 | 3621.1 | 0.0 | 3633.0 | 136.3 | 949.7 | 458.1 | 0.0 | 1544.1 |
| 17 | 0.0 | 0.0 | 3433.2 | 16.4 | 3449.6 | 0.0 | 190.4 | 1438.9 | 0.0 | 1629.2 |
| 26 | 0.0 | 0.0 | 1989.9 | 0.0 | 1989.9 | 96.4 | 315.7 | 1419.4 | 0.0 | 1831.5 |
| 39 | 0.0 | 0.0 | 3045.1 | 437.8 | 3482.9 | 0.0 | 0.0 | 2814.8 | 0.0 | 2814.8 |
| 42 | 0.0 | 0.0 | 3820.7 | 0.0 | 3820.7 | 79.1 | 211.4 | 2200.0 | 0.0 | 2490.5 |
| 4 | 0.0 | 189.1 | 3534.0 | 0.0 | 3723.1 | 642.7 | 1298.8 | 1129.4 | 0.0 | 3071.0 |
| 23 | 0.0 | 0.0 | 3229.3 | 0.0 | 3229.3 | 447.3 | 895.4 | 898.7 | 0.0 | 2241.3 |
| 25 | 0.0 | 16.8 | 3710.0 | 0.0 | 3726.8 | 564.9 | 1271.2 | 839.4 | 0.0 | 2675.5 |
| 33 | 0.0 | 0.0 | 3683.7 | 0.0 | 3683.7 | 358.6 | 575.2 | 2628.6 | 0.0 | 3562.4 |
| 38 | 0.0 | 0.0 | 3945.0 | 0.0 | 3945.0 | 497.3 | 737.0 | 2462.0 | 0.0 | 3696.3 |
| 41 | 0.0 | 464.5 | 4656.9 | 0.0 | 5121.4 | 758.1 | 1628.5 | 948.1 | 0.0 | 3334.6 |

**Supplementary material table S2** Absolute fiber length of dendrite. IT neurons have two dendritic spreading patterns, including IT1 and IT2. IT1 have little apical dendrite in layer 1, colored with yellow. IT2 have much apical dendrite in layer 1, colored with magenta.

| **#** | **Absolute axon length in neocortex (μm)** | | | |
| --- | --- | --- | --- | --- |
|  | **Layer 1** | **Layer 2/3** | **Layer 5** | **Layer 6** |
| 3 | 0.0 | 0.0 | 205.3 | 1081.8 |
| 11 | 0.0 | 0.0 | 189.2 | 216.5 |
| 15 | 95.9 | 275.2 | 2113.6 | 368.8 |
| 19 | 0.0 | 0.0 | 47.0 | 1208.4 |
| 20 | 0.0 | 0.0 | 0.0 | 197.3 |
| 22 | 0.0 | 0.0 | 169.8 | 763.5 |
| 24 | 0.0 | 0.0 | 192.5 | 410.0 |
| 28 | 0.0 | 39.5 | 825.6 | 361.6 |
| 31 | 0.0 | 0.0 | 0.0 | 401.4 |
| 32 | 0.0 | 0.0 | 249.6 | 613.0 |
| 35 | 0.0 | 0.0 | 236.7 | 578.2 |
| 36 | 0.0 | 0.0 | 2612.5 | 2038.6 |
| 5 | 0.0 | 0.0 | 1687.2 | 719.3 |
| 6 | 0.0 | 0.0 | 728.1 | 4169.5 |
| 7 | 0.0 | 0.0 | 629.8 | 953.3 |
| 9 | 0.0 | 0.0 | 225.3 | 1043.3 |
| 12 | 0.0 | 0.0 | 696.9 | 449.5 |
| 13 | 0.0 | 0.0 | 0.0 | 248.8 |
| 16 | 0.0 | 0.0 | 1394.5 | 1012.9 |
| 18 | 0.0 | 0.0 | 160.3 | 253.4 |
| 21 | 0.0 | 0.0 | 2022.8 | 625.8 |
| 27 | 0.0 | 0.0 | 6037.3 | 1486.7 |
| 29 | 0.0 | 0.0 | 213.2 | 249.3 |
| 30 | 0.0 | 0.0 | 0.0 | 268.2 |
| 34 | 0.0 | 0.0 | 554.6 | 822.1 |
| 37 | 0.0 | 47.0 | 2020.4 | 936.1 |
| 1 | 0.0 | 0.0 | 781.9 | 814.5 |
| 2 | 0.0 | 126.4 | 765.0 | 275.6 |
| 8 | 0.0 | 0.0 | 1272.3 | 543.8 |
| 40 | 6.3 | 216.4 | 2781.7 | 1013.8 |
| 10 | 0.0 | 478.3 | 2031.6 | 2141.6 |
| 14 | 0.0 | 0.0 | 417.5 | 1891.7 |
| 17 | 0.0 | 0.0 | 710.6 | 1608.2 |
| 26 | 0.0 | 0.0 | 125.9 | 1101.1 |
| 39 | 0.0 | 0.0 | 4785.8 | 2971.4 |
| 42 | 0.0 | 0.0 | 724.6 | 713.0 |
| 4 | 0.0 | 0.0 | 1586.7 | 824.9 |
| 23 | 0.0 | 0.0 | 186.8 | 1075.5 |
| 25 | 0.0 | 0.0 | 185.8 | 718.2 |
| 33 | 0.0 | 0.0 | 1412.7 | 1144.0 |
| 38 | 0.0 | 0.0 | 2549.9 | 602.6 |
| 41 | 312.8 | 1623.1 | 3380.0 | 1954.2 |

**Supplementary material table S3** Absolute fiber length of local axon around soma in neocortex.

| **#** | **Axon** | | | | | **Basal Dendrite** | | | | | | **Apical dendrite** | | | | |
| --- | --- | --- | --- | --- | --- | --- | --- | --- | --- | --- | --- | --- | --- | --- | --- | --- |
|  | **Branch node** | **End node** | **Total length (μm)** | **Branch quantity** | **Max branch order** | **Quantity** | **Branch node** | **End node** | **Total length (μm)** | **Branch quantity** | **Max branch order** | **Branch node** | **End node** | **Total length (μm)** | **Branch quantity** | **Max branch order** |
| 1 | 56 | 57 | 16.7 | 113 | 18 | 9 | 28 | 37 | 2.9 | 65 | 7 | 61 | 63 | 6.5 | 124 | 26 |
| 2 | 72 | 73 | 22.5 | 145 | 31 | 8 | 24 | 32 | 3.5 | 56 | 5 | 47 | 48 | 6.6 | 95 | 21 |
| 3 | 239 | 241 | 44.3 | 480 | 38 | 8 | 33 | 41 | 4.2 | 74 | 5 | 35 | 36 | 3.6 | 71 | 17 |
| 4 | 197 | 198 | 29.4 | 395 | 37 | 9 | 24 | 33 | 3.7 | 57 | 7 | 26 | 27 | 3.1 | 53 | 15 |
| 5 | 129 | 130 | 31.8 | 259 | 45 | 9 | 33 | 42 | 3.6 | 75 | 6 | 47 | 48 | 5.1 | 95 | 28 |
| 6 | 86 | 87 | 23.1 | 173 | 20 | 9 | 28 | 37 | 2.8 | 65 | 5 | 43 | 44 | 4.7 | 87 | 23 |
| 7 | 111 | 112 | 29.5 | 223 | 21 | 8 | 23 | 31 | 3.5 | 54 | 7 | 54 | 55 | 6.7 | 109 | 27 |
| 8 | 65 | 66 | 17.8 | 131 | 19 | 9 | 18 | 27 | 3.0 | 45 | 5 | 39 | 40 | 5.3 | 79 | 22 |
| 9 | 16 | 17 | 17.4 | 33 | 9 | 8 | 24 | 32 | 3.5 | 56 | 6 | 62 | 64 | 6.9 | 126 | 27 |
| 10 | 89 | 90 | 40.9 | 179 | 18 | 5 | 20 | 25 | 2.3 | 45 | 5 | 27 | 30 | 2.2 | 57 | 16 |
| 11 | 37 | 38 | 20.4 | 75 | 18 | 8 | 20 | 28 | 3.3 | 48 | 4 | 48 | 51 | 6.1 | 99 | 22 |
| 12 | 67 | 69 | 34.6 | 136 | 24 | 8 | 23 | 33 | 3.3 | 56 | 4 | 50 | 53 | 6.7 | 103 | 26 |
| 13 | 14 | 15 | 17.5 | 29 | 11 | 10 | 19 | 29 | 3.2 | 48 | 4 | 36 | 38 | 4.3 | 74 | 26 |
| 14 | 40 | 41 | 24.0 | 81 | 17 | 6 | 27 | 34 | 3.6 | 61 | 6 | 10 | 11 | 1.5 | 21 | 10 |
| 15 | 51 | 52 | 33.6 | 103 | 17 | 8 | 28 | 36 | 3.6 | 64 | 6 | 46 | 50 | 6.5 | 96 | 24 |
| 16 | 22 | 23 | 20.1 | 45 | 11 | 7 | 11 | 19 | 2.1 | 30 | 4 | 45 | 47 | 6.0 | 92 | 16 |
| 17 | 27 | 28 | 18.3 | 55 | 10 | 4 | 31 | 37 | 3.4 | 68 | 7 | 18 | 19 | 1.6 | 37 | 12 |
| 18 | 107 | 108 | 42.6 | 215 | 20 | 11 | 16 | 29 | 3.9 | 45 | 4 | 35 | 36 | 5.3 | 71 | 22 |
| 19 | 9 | 10 | 17.2 | 19 | 7 | 8 | 22 | 34 | 3.5 | 56 | 4 | 25 | 29 | 3.4 | 54 | 12 |
| 20 | 10 | 11 | 16.2 | 21 | 9 | 7 | 35 | 44 | 4.4 | 79 | 6 | 41 | 44 | 5.6 | 85 | 17 |
| 21 | 35 | 36 | 29.8 | 71 | 15 | 10 | 21 | 32 | 3.9 | 53 | 5 | 53 | 58 | 6.8 | 111 | 23 |
| 22 | 9 | 10 | 14.6 | 19 | 8 | 8 | 16 | 26 | 3.0 | 42 | 4 | 35 | 39 | 5.3 | 74 | 24 |
| 23 | 97 | 98 | 47.1 | 195 | 20 | 5 | 22 | 31 | 3.2 | 53 | 6 | 21 | 22 | 2.2 | 43 | 18 |
| 24 | 4 | 5 | 15.2 | 9 | 5 | 8 | 13 | 24 | 2.1 | 37 | 4 | 38 | 42 | 4.7 | 80 | 16 |
| 25 | 76 | 77 | 49.2 | 153 | 19 | 7 | 16 | 28 | 3.7 | 44 | 4 | 21 | 22 | 2.7 | 43 | 16 |
| 26 | 19 | 20 | 15.8 | 39 | 7 | 7 | 16 | 23 | 2.0 | 39 | 5 | 12 | 15 | 1.8 | 27 | 9 |
| 27 | 107 | 108 | 49.1 | 215 | 19 | 7 | 26 | 34 | 3.7 | 60 | 6 | 69 | 73 | 8.0 | 142 | 26 |
| 28 | 22 | 23 | 27.2 | 45 | 9 | 7 | 31 | 40 | 4.6 | 71 | 5 | 43 | 47 | 5.4 | 90 | 23 |
| 29 | 17 | 18 | 20.5 | 35 | 11 | 9 | 27 | 36 | 4.6 | 63 | 6 | 49 | 52 | 6.7 | 101 | 20 |
| 30 | 9 | 10 | 16.4 | 19 | 9 | 8 | 29 | 39 | 3.7 | 68 | 6 | 37 | 38 | 4.2 | 75 | 20 |
| 31 | 12 | 13 | 16.9 | 25 | 9 | 7 | 28 | 37 | 3.0 | 65 | 7 | 42 | 46 | 4.6 | 88 | 26 |
| 32 | 7 | 8 | 16.4 | 15 | 5 | 8 | 24 | 32 | 3.1 | 56 | 5 | 51 | 56 | 6.2 | 107 | 24 |
| 33 | 107 | 108 | 67.8 | 215 | 16 | 5 | 26 | 31 | 3.7 | 57 | 6 | 27 | 28 | 3.6 | 55 | 17 |
| 34 | 35 | 36 | 29.5 | 71 | 12 | 7 | 26 | 33 | 3.9 | 59 | 6 | 44 | 46 | 6.3 | 90 | 23 |
| 35 | 12 | 13 | 17.7 | 25 | 7 | 7 | 23 | 30 | 3.2 | 53 | 5 | 49 | 50 | 5.9 | 99 | 28 |
| 36 | 50 | 51 | 30.4 | 101 | 14 | 7 | 25 | 36 | 3.5 | 61 | 6 | 49 | 51 | 5.5 | 100 | 22 |
| 37 | 40 | 41 | 26.4 | 81 | 11 | 5 | 30 | 38 | 3.7 | 68 | 7 | 60 | 62 | 7.5 | 122 | 25 |
| 38 | 54 | 55 | 31.8 | 109 | 16 | 5 | 34 | 39 | 3.9 | 73 | 6 | 36 | 37 | 3.7 | 73 | 17 |
| 39 | 49 | 50 | 32.4 | 99 | 16 | 5 | 35 | 40 | 3.5 | 75 | 7 | 31 | 33 | 2.8 | 64 | 15 |
| 40 | 72 | 75 | 36.4 | 147 | 22 | 10 | 21 | 33 | 3.7 | 54 | 4 | 53 | 62 | 6.6 | 115 | 22 |
| 41 | 69 | 70 | 51.6 | 139 | 15 | 9 | 31 | 40 | 5.1 | 71 | 9 | 27 | 28 | 3.3 | 55 | 13 |
| 42 | 21 | 22 | 16.7 | 43 | 9 | 6 | 30 | 39 | 3.8 | 69 | 5 | 23 | 25 | 2.5 | 48 | 14 |

**Supplementary material table S4** Morphological parameters of 42 neurons.


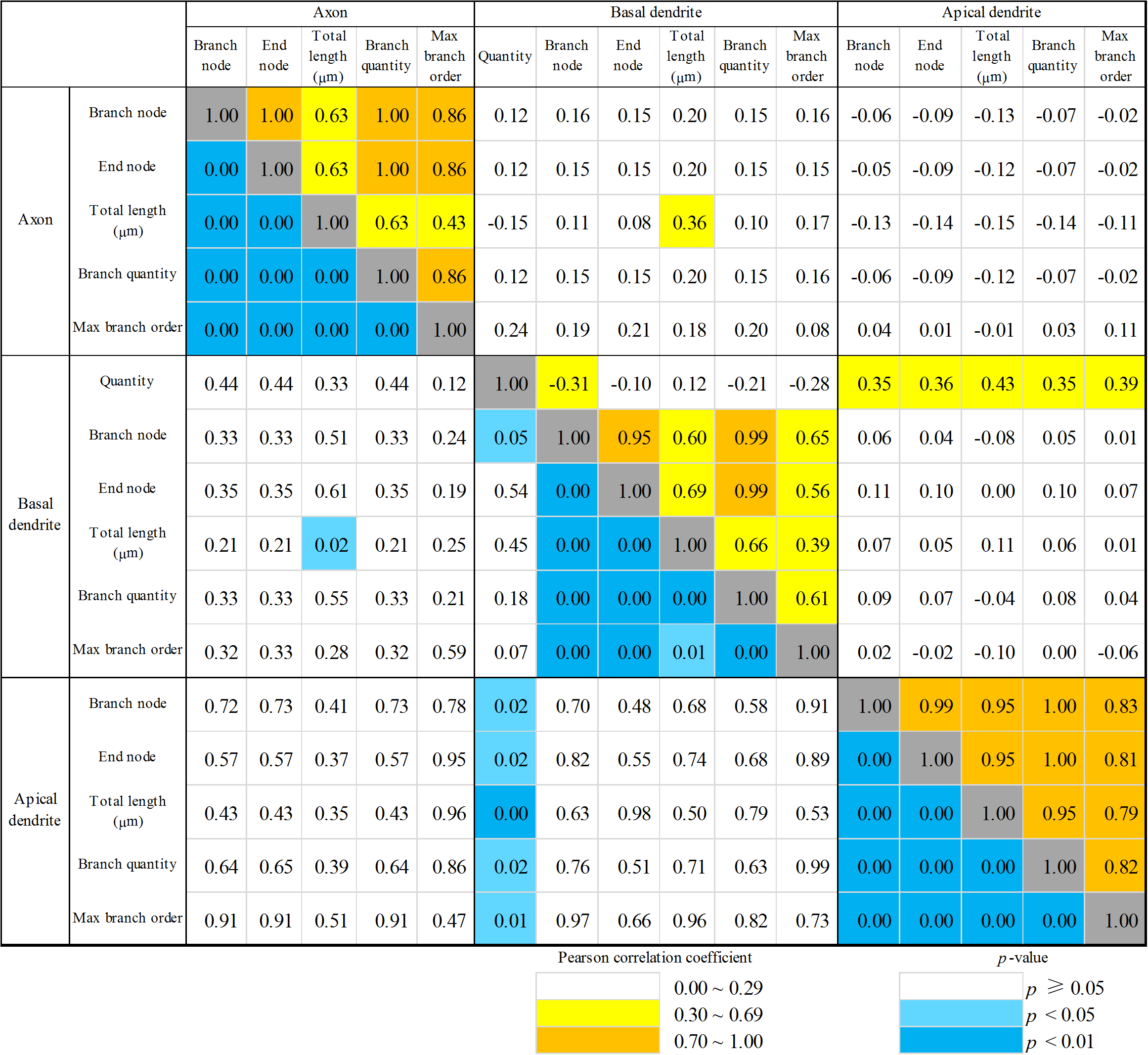


**Supplementary material Figure S5** The upper triangle matrix records the Pearson correlation coefficient, and lower triangular matrix records the *p*-value of the Pearson correlation coefficient of the corresponding position. Because the Pearson correlation coefficient matrix and matrix of *p*-value are symmetry, the data of the symmetric position in the matric is corresponding. The correlation strengths are labelled with different color shades.


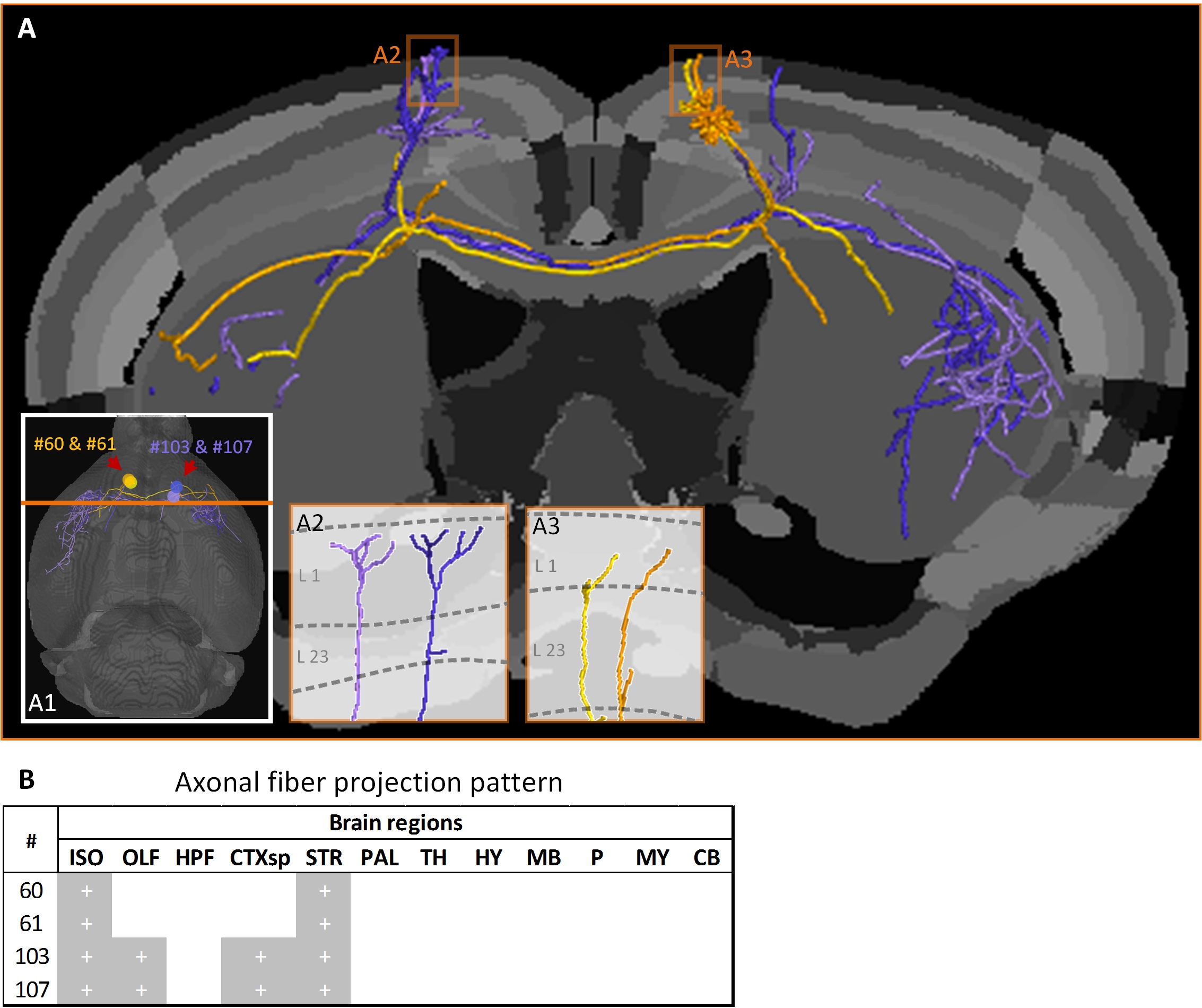


**Supplementary material Figure S6** The validity of the two dendritic spreading pattern of IT neurons. (A) Four reconstructed neurons morphology in coronal view. The two neuron with IT_1_ dendritic spreading pattern are colored with yellow. We only reconstructed the axon major skeleton (brightest fiber) to show it is contralateral projection pattern. The two neuron with IT_2_ dendritic spreading pattern are colored with magenta. (A1) the horizontal view of the four neurons in brain. The orange line indicates the coronal plane location in axial direction. The soma of four reconstructed neurons are indicated by the colored dots. (A2) The apical dendrite morphology of two neurons with IT_2_ dendrite spreading pattern. (A3) The apical dendrite morphology of two neurons with IT_1_ dendrite spreading pattern. Grey dash line is the layer border. (B) The axonal fiber projection pattern of four neurons shown in (A).


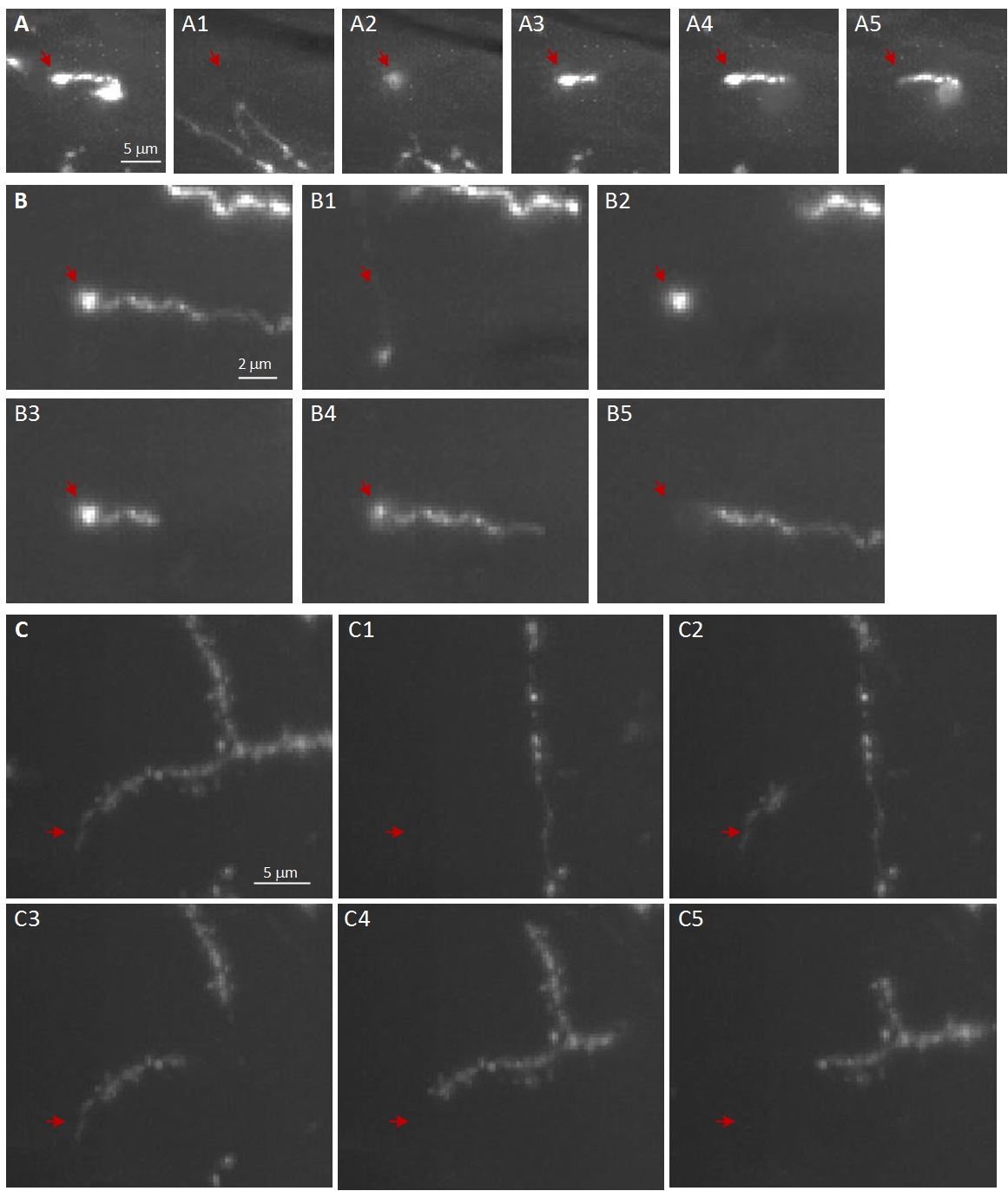


**Supplementary material Figure S7** The images of axonal and dendritic fiber terminal from the neuron we choose to reconstruct. (A) The 10 μm maximum projection an axonal fiber terminal. (A1-A5) The continue slices of a 5 μm maximum projection image to show the morphology of axonal fiber terminal when we tracing neurons. (A1- A5) are overlapped in axial direction to display terminal morphology better. (B) The 40 μm maximum projection image of an axonal fiber terminal. (B1-B5) The continue slices of 10 μm maximum projection image. (C) The 40 μm maximum projection image of a dendritic fiber terminal. (C1-C5) The continue slices of 20 μm maximum projection image.

**Supplementary information**

**Local axonal fiber analysis**

Local axonal pattern sometimes also suggests different morphology[^1^](#_ENREF_1)^,^[^2^](#_ENREF_2). We calculate the axonal fiber around soma in the neocortex using the same method as dendrite. Instead of measure the relative fiber length in cortex, we use the absolute length to present the local axonal fiber characters, the data are present in supplementary materials table 3. We compare the local axonal fiber length between IT vs PT, PT1 vs PT2. IT1 vs IT2. There only significant difference between IT and PT in layer 6 (Wilcoxon rank sum test, p=0.0036).

**Self-correlation of morphological statistic parameters from different neuron parts**

Morphological statistic parameters are powerful to descript neuron morphology[^3-5^](#_ENREF_3). We use sixteen basic morphological statistic parameters to analyze reconstructed neuron morphologies. We find that morphological statistic parameters show correlation in the same part of neurons. And the correlations between different neurons parts are weak. Specifically, from supplementary material figure 1, five parameters from axon have strong or medium correlation with the parameters form axon, but have weak correlation with parameters from basal or apical dendrites. The same situation also occurs on the basal dendrites and apical dendrite, except for the correlation between total length from axon and basal dendrite, and the correlation between basal dendrite quantity and the parameters from apical and basal dendrite. In detail, total length of axon has a medium correlation with total length of basal dendrite, which is the only parameters from axon correlate with dendrite parameters. On the other hand, stem quantity is the unique parameter that only calculated from basal dendrite. Because, from our reconstructed neuron morphology, the axon and apical dendrite have only one stem growing from soma. While, there are more than one stem of basal dendrite growing form soma. Surprisingly, five parameters from apical dendrite all have medium correlation with the stem quantity of basal dendrite.

**Morphological parameters**

The basic morphology parameters of 42 neurons are calculated using Neurolucida Explore (Neurolucida Explorer, Version 2.70.1, MBF Bioscience, Williston, VT). We calculate five morphological parameters of the axon and apical dendrite, and 6 parameters of basal dendrite. The morphological parameters are defined as follows: the branch node is the quantity of fiber branch points; the end node is the quantity of fiber terminal tips; the total length is the total length for a given fiber type; the branch quantity is calculated for a given fiber type; the quantity refers to the number of basal dendritic stem growing from soma; the max branch order is the maximum centrifugal order for a given fiber type.

**References**

1 Staiger, J. F., Loucif, A. J., Schubert, D. & Mock, M. Morphological Characteristics of Electrophysiologically Characterized Layer Vb Pyramidal Cells in Rat Barrel Cortex. *PloS one* **11**, e0164004, doi:10.1371/journal.pone.0164004 (2016).

2 Oberlaender, M. *et al.* Three-dimensional axon morphologies of individual layer 5 neurons indicate cell type-specific intracortical pathways for whisker motion and touch. *Proceedings of the National Academy of Sciences of the United States of America* **108**, 4188-4193, doi:10.1073/pnas.1100647108 (2011).

3 Uylings, H. B. & van Pelt, J. Measures for quantifying dendritic arborizations. *Network* **13**, 397-414 (2002).

4 Guo, C. *et al.* Single-axon level morphological analysis of corticofugal projection neurons in mouse barrel field. *Sci Rep* **7**, 2846, doi:10.1038/s41598-017-03000-8 (2017).

5 Cervantes, E. P., Comin, C. H., Junior, R. M. C. & Costa, L. D. F. Morphological Neuron Classification Based on Dendritic Tree Hierarchy. *Neuroinformatics* **17**, 147-161, doi:10.1007/s12021-018-9388-7 (2019).
